# The Therapeutic Value of Recent Synthetic Analgesics—Their Benefits and Attendant Risks

**Published:** 1898-12

**Authors:** 


### THE THERAPEUTIC VALUE OF RECENT SYNTHETIC ANALGESICS—THEIR BENEFITS AND ATTENDANT RISKS.

Phillips (*British Medical Journal*, October 8, 1898), at the last meeting of the British Medical Association, gave a summary of the actions of the recent analgesics, of which the following is an abstract: It is doubtful if, with one exception, the recent synthetic analgesics even approximate in their therapeutic value to the older ones, such as antipyrin, antifebrin, phenacetin, and exalgin. They group themselves into three main divisions, according as they are derived from chinoline, aniline, or salicylic acid. One alone is derived from chinoline—namely, analgene. The derivatives of aniline are europhen, methylene blue, pyoktanin, agathin, malakin, neuroidin, lactophenin, apolysin, and citrophen; also a subgroup, consisting of pyramidon, salipyrin, tolipyrin, tolisall, and ferripyrin, compounds of antipyrin; and another subgroup, consisting of phenocoll, which is derived from phenacetin.

Salophen, salocoll, salipyrin, tolisall, malakin, and agathin are derived from both aniline and salicylic acid.

Analgene is not very powerful, but has the advantages of but slight toxicity, of tastelessness, and of easy solubility in acid media, or that being readily absorbed from the stomach, its action is rapidly produced. It is of special value in the treatment of painful affections of children. It has been proved of value in many diseases, among which are spinal caries and hip-joint disease. It has sometimes succeeded after antipyrin and phenacetin have failed. Its great evil depends on the destructive action it exerts on the red blood-corpuscles resulting in urobilinuria. Unpleasant accidents—nausea, vomiting, diarrhea, tremor, vertigo, and rashes—rarely occur.

Europhen is a powerful analgesic; it is said to have the activity of twice its weight of antipyrin. But it tends to interfere with the respiratory processes and to weaken the heart's action, and produce cyanosis and collapse. A negative advantage it has, in that it does not induce changes in the blood or affect the kidneys. It has proved of especial service in the pain of orchitis. Although its toxicity, in carefully regulated doses, can not be called great, yet it always produces extremely profuse perspiration, frequently a subnormal temperature, and occasionally cyanosis. The great disadvantage is the uncertainty of its action; it should therefore be given, in the first instance, in a tentative manner.

Methylene blue is a distinctly useful analgesic, particularly in functional neuralgias and in all kinds of nervous headache. It, however, changes hemoglobin into methemoglobin and causes irritation of the stomach, leading to vomiting and diarrhea, and also of the urinary tract. In large doses it has caused muscular paresis, loss of sensibility, and dyspnea.

Pyoktanin is not of sufficient therapeutic value to need more than a mere reference.

Agathin is said to be both slow in its action and unreliable in its effect. It has caused vomiting, diarrhea, insomnia, vertigo, unconsciousness, and even increase of pain. It is probably not worthy a place among valued analgesics.

The compounds of antipyrin—pyramidon, salipyrin, tolisall, and ferripyrin—have as analgesics merely a weak action of their antipyrin element. They are harmless, but not, as a rule, sufficiently powerful for the treatment of urgent pain.

Those which are derived from salicylic acid as well as antipyrin—namely, salipyrin and tolisall—are of use in painful states of rheumatic origin, but otherwise they are comparatively unimportant.

Malakin, being only slightly soluble in the ordinary menstrua, is slow in its action, very much slower than phenacetin, antifebrin and antipyrin, and is in every way much inferior to them; with the exception, however, that it may be given for a long period without disordering digestion. It has been found of distinct service in acute rheumatism. It has no deleterious action on the blood or kidneys, being indeed practically free from risk.

Neuroidin is another remedy feeble in action, but harmless in doses sufficiently large to elicit its analgesic effect. It is, however, uncertain, and must be considered greatly inferior to phenacetin and antipyrin.

Lactophenin appears to be more active as a mere sedative to the nervous system (relieving irritability, restlessness, and depression) than as a pure analgesic. It is comparatively free from risk, although in medicinal doses it has produced vomiting, coldness, cyanosis, and collapse. But its great disadvantage is that it is inconstant in its analgesic effect; it has been known to fail completely in relieving the pain of neuritis and of intercostal neuralgia.

Citrophen has not so far been much used as an analgesic. Its applications are much the same as those of phenacetin, but its attendant risks are greater, while its benefits are much less distinct. It has exerted in medicinal doses a grave toxic effect on the blood and on the kidneys. It probably ranks very much below phenacetin.

Apolysin closely resembles phenacetin in its effects. It is said to be more powerful, but likewise more toxic, as it is more soluble than phenacetin and more prompt. It has been of especial benefit in migraine. It is well tolerated in large doses, continued over a long period of time without producing intestinal or renal irritation, or, indeed, any material injurious result. Its toxic effects are especially liable to occur if it is given on an empty stomach, or when that organ is producing an excessive amount of secretion. It is a remedy which may prove a valuable supplement to the phenacetin-antipyrin-exalgin group.

Phenocall-hydrochloride is a derivative of phenacetin, and is said to be as powerful an analgesic as it, and being more soluble, it acts more quickly. But it is not so safe. Its special use has been in neuralgias brought on by cold—that is, of rheumatic origin. But in children and debilitated subjects, more especially in cases of advanced phthisis, it has caused alarming collapse with cyanosis. It is, however, a substance which may take an important place in the future as a means of relieving painful states generally. The salicylate of phenocoll or salocoll has no advantage over phenocoll, except that which is due to the action of its salicylic acid element.

Salophen is probably the best of the recent synthetic analgesics. Like salol, it is not decomposed until it reaches the intestine, so it does not produce any gastric disturbance as do the salicylates. Its toxicity is very slight, and is apparently due, when given in very large doses, to the salicylic acid it contains, but in medicinal doses it does not even produce the tinnitus and headache characteristic of the action of the salicylates. It has been of benefit in painful states of all sorts, but of special value in the neuralgias of children. When it is used to relieve the pains of influenza it is necessary to continue it for several days after the pains have been relieved.

Conclusions.—Salophen, phenocoll-hydrochloride, apolysin, and methylene blue are substances of high therapeutic value, and without attempting to arrange them in order, salophen appears to be of exceptional promise. Agathin, being slow, unreliable, and even dangerous in its action, should be avoided. Some hesitation should be exercised in employing analgene and citrophen, because of their toxic action on the blood; in using europhen and lactophenin, because of their inconstancy and tendency to produce collapse, and in choosing malakin, because of its tardy action. Pyoktanin, ferripyrin, pyramidon, salipyrin, tolipyrin, tolisoll, and even salocoll, although they may be well and safely used as substitutes for better remedies, are unnecessary.—*University Medical Journal.*

